# Long-term renal and overall survival of critically ill patients with acute renal injury who received continuous renal replacement therapy

**DOI:** 10.1080/0886022X.2017.1398667

**Published:** 2017-12-04

**Authors:** Lingping Wu, Ping Zhang, Yi Yang, Hua Jiang, Yongchun He, Chunping Xu, Huijuan Yan, Qi Guo, Qun Luo, Jianghua Chen

**Affiliations:** a Kidney Disease Center, The First Affiliated Hospital, Medical College, Zhejiang University, Hangzhou, PR China;; b Department of Nephrology, Ningbo No. 2 Hospital, Ningbo, PR China

**Keywords:** Acute kidney injury, continuous renal replacement therapy, critically ill, long-term survival

## Abstract

**Background:** Acute kidney injury (AKI) is associated with the increased short-term mortality of critically ill patients on continuous renal replacement therapy (CRRT). The aim of this research was to evaluate the association of kidney function at discharge with the long-term renal and overall survival of critically ill patients with AKI who were on CRRT in an intensive care unit (ICU).

**Methods:** We retrospectively collected data for critically ill patients with AKI who were admitted to ICU on CRRT at a tertiary metropolitan hospital in China between 2008 and 2013. The patients were followed up to their death or to 30 September 2016 by telephone.

**Results:** A total of 403 patients were enrolled in this study. The 1-, 3- and 5-year patient survival rates were 64.3 ± 2.4, 55.8 ± 2.5 and 46.3 ± 2.7%, respectively. In multivariate analysis, age, sepsis, decreased renal perfusion (including volume contraction, congestive heart failure, hypotension and cardiac arrest), preexisting kidney disease, Apache II score, Saps II score, vasopressors and eGFR <45 mL/min/1.73 m^2^ at discharge were independent factors for worse long-term patient survival. And age, preexisting kidney disease, Apache II score, mechanical ventilation (MV) and eGFR <45 mL/min/1.73 m^2^ at discharge were also associated with worse renal survival.

**Conclusions:** This study showed that impaired kidney function at discharge was shown to be an important risk factor affecting the long-term renal survival rates of critically ill patients with AKI. An eGFR <45 mL/min/1.73 m^2^ was an independent risk factor for decreased overall survival and renal survival.

## Introduction

Acute kidney injury (AKI) is characterized by the rapid loss of renal function, resulting in a number of complications, including fluid imbalance, metabolic acidosis and uremia [1], and it occurs quite commonly in hospitalized patients. AKI is also a common clinical problem encountered with critically ill patients and is generally related to an increase in morbidity and mortality [2]. According to epidemiological investigations, AKI affects an estimated 13–18% of hospitalized patients [3]. However, the incidence of AKI in critically ill patients varies from 30 to 60%, and it is associated with increasing in-hospital mortality in critically ill patients requiring renal replacement therapy (RRT) [4–6]. Numerous clinical studies indicate the relationships between AKI and long-term mortality, the development of chronic kidney disease (CKD), as well as the eventual progression to end-stage renal disease (ESRD) [7–12]. However, there are also some patients with a complete recovery from AKI may be confronted with adverse long-term outcomes [13–16], and those with little recovery or partial recovery can be faced with an aggravation of predisposition [17–20].

Being a worldwide public health problem, it is indicated that AKI can lead to CKD in 10% of critically ill patients [21]. In addition, it is declared that need for RRT increased the likelihood of the progression to CKD by 500-fold [22]. Wu et al. revealed the long-term survival of 4393 AKI patients after surgery (follow-up of 4.76-years) and found hazard ratios (HRs) of 1.94, 2.64 and 3.28 for patient mortality associated with AKI, CKD and AKI on CKD, respectively [17].

Existing analyses primarily focused on assessing in-hospital mortality in patients with AKI demanding continuous renal replacement therapy (CRRT) [23]. Meanwhile, renal function at discharge may be associated with increased mortality at follow-up, which has not been concerned much [24]. The aim of this study was to research on the relation between renal function at discharge and long-term renal survival and overall long-term mortality of critically ill patients diagnosed with AKI and treated with CRRT in the intensive care unit (ICU).

## Patients and methods

### Patients

This study was conducted retrospectively in ICU at the First Affiliated Hospital, Medical College, Zhejiang University from 1 January 2008 to 31 December 2013. The patients were survivors at discharge among those in the ICU, who diagnosed with AKI and accepted CRRT. The patients were excluded for the following reasons: on RRT or kidney transplantation before ICU admission; died in hospital; incomplete data; lack of follow-up.

#### AKI criteria

In this study, the AKI diagnostic criteria referred to KDIGO AKI Guideline Work Group: an absolute increase in serum creatinine of ≥0.3 mg/dl (≥26.4 µmol/L), a percentage increase in serum creatinine of ≥50% (1.5-fold from baseline), or a reduction in urine output (documented oliguria of <0.5 mL/kg/h for >6 h) [25].

### Data collection

#### Demographic data and clinical data

Age, gender, weight, urine output before CRRT, medical history, causes of AKI, presence of comorbidities, the use of vasopressors, requirements for mechanical ventilation (MV), acute physiology and chronic health evaluation (APACHE) II score [26], simplified acute physiology score (SAPS II) [27], length of hospital stay, length of ICU stay, length of CRRT stay and CRRT therapeutic dose of all patients.

#### Laboratory data

Routine blood parameters, liver function, kidney function, blood gases and blood electrolytes before CRRT, the liver function and kidney function at discharge.

#### Follow-up information

The patients were followed up to their death or to 30 September 2016 by telephone. The renal survival was considered as the patients without RRT or transplantation.

### Groups

According to the modification of diet in renal disease (MDRD) formula: estimated glomerular filtration rate (eGFR) (mL/min1.73 m^2^) = 186 * (Scr) − 1.154 * (age) − 0.203* (0.742 female), the kidney function at discharge was arbitrarily defined with per eGFR category for the estimation of GFR. As the eGFR levels at discharge, patients were categorized into five groups: the first group was defined as eGFR ≥60 mL/min per 1.73 m^2^, the second category was defined as eGFR of 45–59 mL/min per 1.73 m^2^, the third category was defined as eGFR of 30–44 mL/min per 1.73 m^2^, the fourth category was defined as eGFR of 15–29 mL/min per 1.73 m^2^ and the last category was defined as eGFR <15 mL/min per 1.73 m^2^ (including RRT) at discharge.

### Statistical analyses

SPSS version 22.0 (SPSS Inc., Chicago, IL) was used for all statistical analyses. Continuous normal data were expressed as the mean ± standard deviation (SD). Non-normal distribution of measurement data were expressed as the median and range. Categorical data were expressed as the number of cases and percentages. Patient survival curves and renal survival curves were generated according to each eGFR category by the Kaplan–Meier method and a log-rank test was employed to analyze statistical differences among groups. Cox proportional hazard survival model was adopted to evaluate independent predictors for long-term mortality. When constructing the Cox multivariate model, univariate with *p* values <.2 were used [28]. Statistical significance was defined as a *p* values <.05. Data were presented as HRs with 95% confidence intervals (CI).

## Results

### Baseline patient characteristics

**Figure 1.**
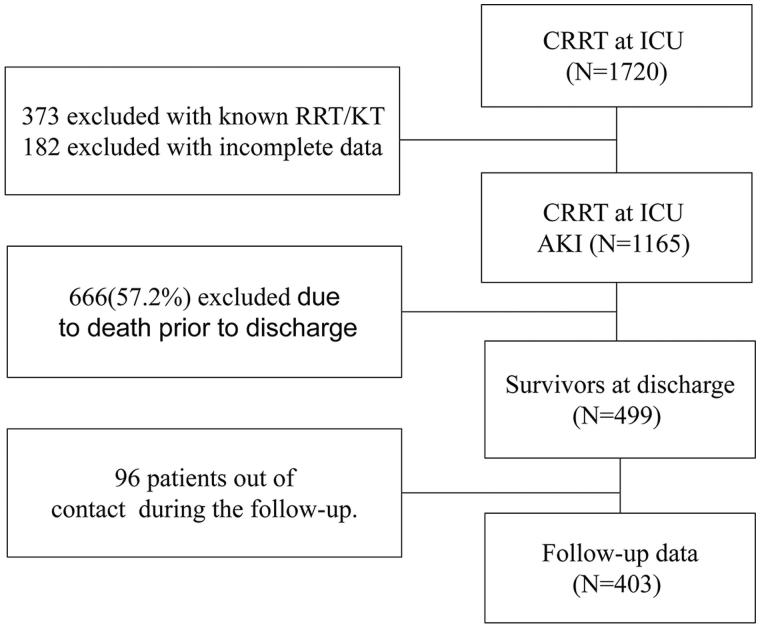
Patients selection scheme.

There were 1720 patients who received CRRT in the ICU between 2008 and 2013 (Figure 1). Among those, 373 patients were excluded because they were on RRT or had received a kidney transplant before ICU admission and 182 patients were excluded because of incomplete data. Of the 1165 patients who were diagnosed with AKI and were treated with CRRT in the ICU, 666 patients (57.2%) died in the hospital and the remaining 499 patients were alive at discharge. Among the 499 patients, 96 patients were lack of follow-up. The remaining 403 patients in total were enrolled in this study (Figure 1).

The main characteristics of the 403 patients analyzed are summarized in Table 1. Among those, 66.5% (268/403) was male and the age was 60.8 ± 17.8 (17–95)years old. Median length of CRRT was 6 d. The median Apache II score was 23, and the median length of survival was 1133 (1–2975) d (Table 1).

**Table 1.** Baseline characteristics at discharge of the 403 patients.

Variable	Mean ± SD/median(range)
Age (years)	60.8 ± 17.8
Gender (male, %)	268 (66.5%)
Length of hospital stay (days)	26 (2–181)
Length of ICU stay (days)	10 (1–161)
Length of CRRT (days)	6 (1–149)
Serum albumin (g/L)	31.1 ± 6.3
Total bilirubin (µmol/L)	23.0 (3.0–638.0)
Serum creatinine (µmol/L)	270.0 (40.0–2069.0)
Urea (mmol/L)	20.6 (3.4–70.7)
Hemoglobin (g/L)	97.3 ± 27.9
Bicarbonate (mmol/L)	19.8 ± 6.7
Lactate level (mmol/L)	1.7 (0.3–15)
24 h urine output (mL)	100 (0–1000)
Apache II score	23 (12–63)
Saps II score	49.3 ± 15.5
Serum albumin at discharge (g/L)	33.7 (20.4–55.0)
Serum creatinine at discharge (µmol/L)	134.0 (38.0–1256.0)
Urea at discharge (mmol/L)	10.6 (2.1–55.8)
Vasopressors (*n*, %)	175 (43.4%)
Mechanical ventilation (*n*, %)	207 (51.4%)
Dose of CRRT (mL/kg.h)	50.0 ± 13.7
Length of survival (days)	1133 (1–2975)

### Causes of AKI

The causes of AKI for these 403 cases are shown in Table 2. Sepsis (38.5%) was the primary cause of AKI, followed by decreased renal perfusion (28.8%) and surgical cause (15.9%) (Table 2).

**Table 2.** The causes of AKI (*n* = 403).

Cause of AKI	*N* (%)
Decreased renal perfusion (including volume contraction, congestive heart failure, hypotension and cardiac arrest)	116 (28.8%)
Sepsis	155 (38.5%)
Surgical	64 (15.9%)
Others	68 (16.9%)

The most common comorbidities of 403 patients are summarized in Table 3. Respiratory diseases, hypertension and cardiovascular diseases occupy the greatest proportion. A percentage of 26.6 (107/403) was diagnosed with preexisting CKD (Table 3).

**Table 3.** The comorbidities of patients (*n* = 403).

Co-morbidities	*N* (%)
Respiratory disease	134 (33.3%)
Hypertension	124 (30.8%)
Cardiovascular disease	113 (28.0%)
Preexisting CKD	107 (26.6%)
Diabetes mellitus	69 (17.1%)
Neoplasm	62 (15.4%)
Liver disease	55 (13.6%)
Neurological disease	26 (6.5%)
Peripheral vascular disease	21 (5.2%)

### Groups

The patients were categorized into five groups according to the eGFR level. There were 135(33.5%), 32(7.9%), 51(12.7%), 56(13.9%) and 129(32.0%) patients in the group defined as eGFR ≥60 mL/min, 45–59 mL/min, 30–44 mL/min, 15–29 mL/min and <15 mL/min, respectively (Table 4).

**Table 4.** Groups according to eGFR levels at discharge (*n* = 403).

Group	*N* (%)
eGFR ≥60 mL/min	135 (33.5%)
eGFR45–59 mL/min	32 (7.9%)
eGFR30–44 mL/min	51 (12.7%)
eGFR15–29 mL/min	56 (13.9%)
eGFR <15 mL/min	129 (32.0%)

### Long-term patient survival

**Figure 2.**
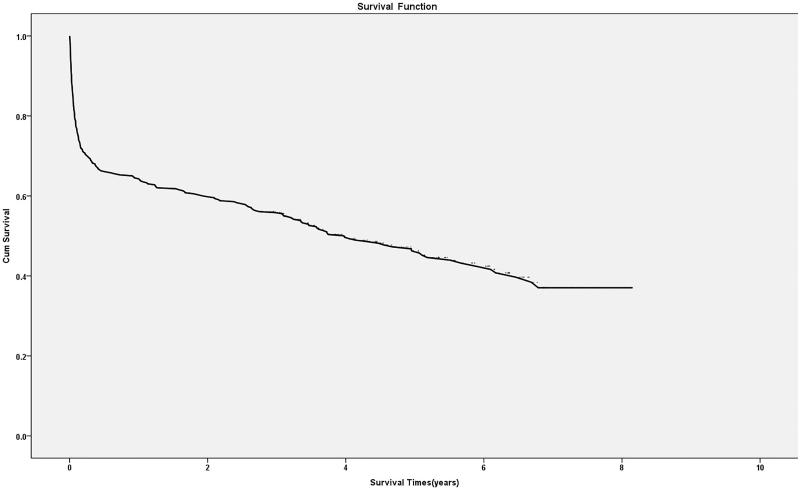
Kaplan–Meier curves for overall survival after hospital discharge.

**Figure 3.**
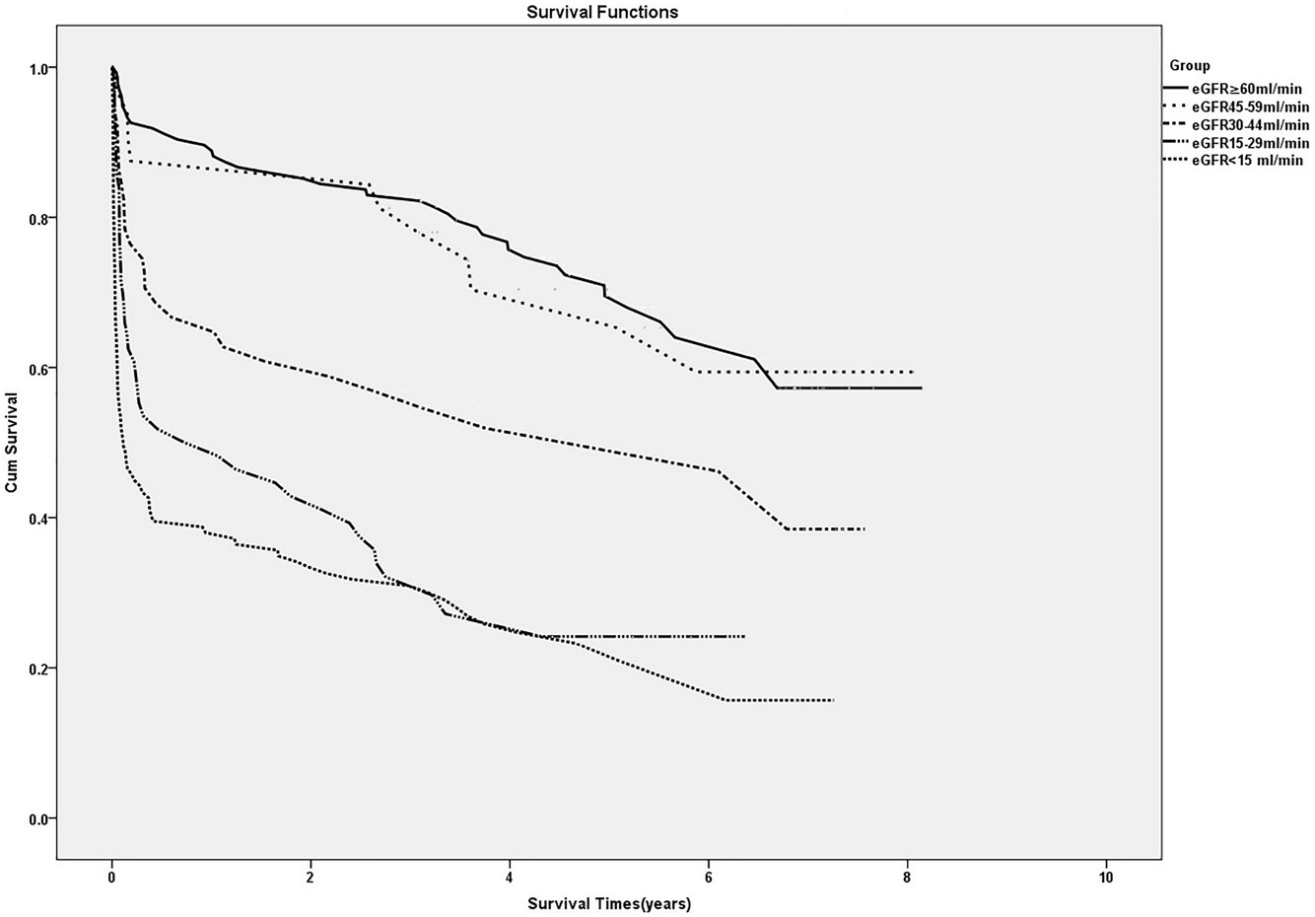
Kaplan–Meier curves for survival after hospital discharge according to renal function.

By 30 September 2016, 53.8% (217/403) patients had died, and the rest of them were still alive. The cumulative survival rates were 64.3 ± 2.4% in the first year, 55.8 ± 2.5% in the third years and 46.3 ± 2.7% in the fifth years (Figure 2). Kaplan–Meier curves for survival indicated that long-term survival was tightly relevant to the degree of kidney dysfunction at discharge (Figure 3). The long-term survival of patients with eGFR30–44 mL/min/1.73 m^2^, with eGFR 15–29 mL/min/1.73 m^2^ and those with eGFR <15 mL/min/1.73 m^2^ at discharge were much lower compared with that of the patients with eGFR ≥60 mL/min/1.73m^2^, according to a log-rank test (*p* =  .001, *p* < .001 and *p* < .001, respectively).

Multivariate proportional hazard regression analysis identified that age, the causes of AKI (sepsis and decreased renal perfusion), a preexisting kidney disease, Apache II score, Saps II score, vasopressors and the eGFR <45 mL/min/1.73 m^2^ groups at discharge were all associated with decreased long-term patient survival (eGFR 30–44 mL/min/1.73m^2^, HR 2.26 [95% CI, 1.36–3.74]; eGFR 15–29 mL/min/1.73 m^2^, HR 4.89 [95% CI, 3.03–7.89]; eGFR <15 mL/min/1.73 m^2^ and HR 5.67 [95% CI, 3.70–8.68]) (Table 5).

**Table 5.** Multivariate analysis of the variables associated with overall survival (*n* = 403).

Variable	HR (95% CI)	*p*
Age	1.03 (1.02–1.04)	<.001*
Gender	0.80 (0.60–1.07)	.135
The cause of AKI		
Sepsis	1.70 (1.05–2.73)	.030*
Decreased renal perfusion	1.67 (1.01–2.73)	.044*
Surgical	1.27 (0.73–2.21)	.395
Others	–	–
Co-morbidities		
Neoplasm	1.04 (0.71–1.53)	.836
Diabetes	1.44 (0.98–2.14)	.066
Hypertension	1.38 (0.99–1.92)	.058
Pre-CKD	1.49 (1.10–2.03)	.010*
Apache II score	1.03 (1.01–1.06)	.005*
Saps II score	1.03 (1.01–1.04)	<.001*
Vasopressors	1.75 (1.30–2.35)	<.001*
Mechanical ventilation	1.04 (0.99–1.08)	.079
Renal function at hospital discharge		
eGFR ≥60 mL/min	–	–
eGFR45–59 mL/min	1.06 (0.52–2.14)	.874
eGFR30–44 mL/min	2.26 (1.36–3.74)	.002*
eGFR15–29 mL/min	4.89 (3.03–7.89)	<.001*
eGFR <15 mL/min	5.67 (3.70–8.68)	<.001*

*Significant at *p* < .05 level.

### Renal survival

**Figure 4.**
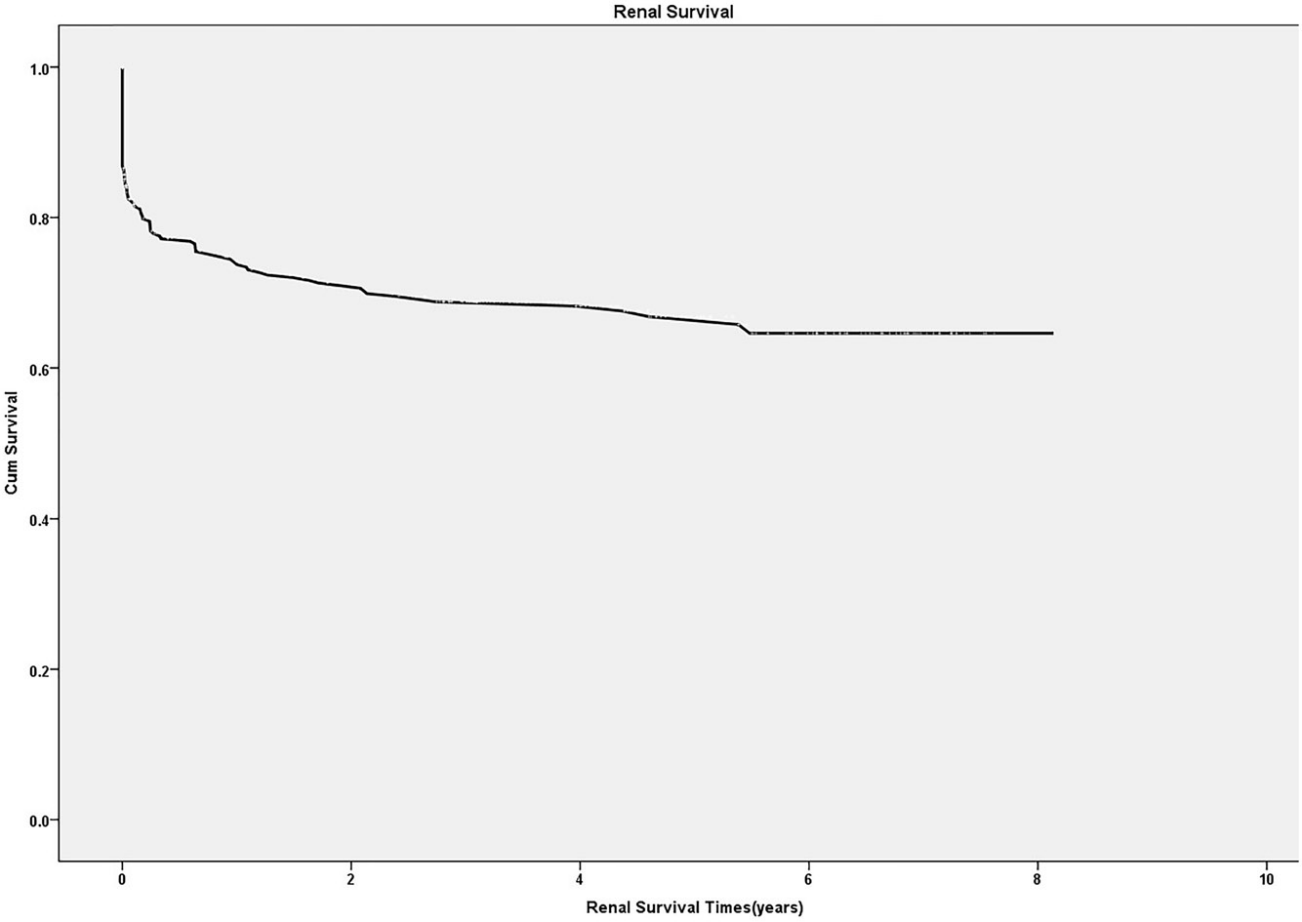
Kaplan–Meier curves for renal survival after hospital discharge.

**Figure 5.**
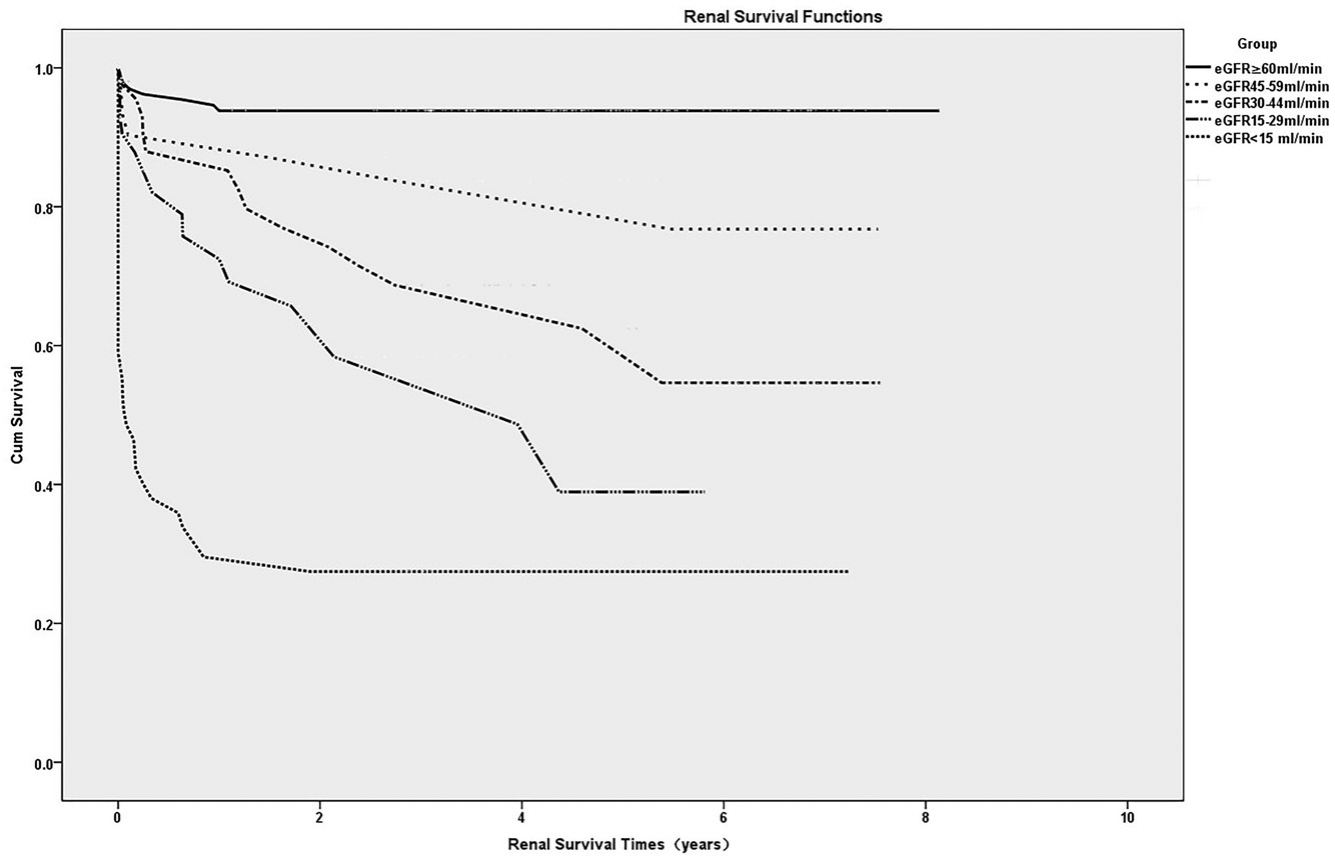
Kaplan–Meier curves for renal survival after hospital discharge according to renal function.

Renal survival rates after hospital discharge in the first, third and fifth year were 74.4 ± 2.3%, 68.8 ± 2.6% and 66.8 ± 2.7%, respectively (Figure 4). A Kaplan–Meier analysis for renal survival after hospital discharge revealed that the renal survival rates of patients with eGFR30–44 mL/min/1.73 m^2^, eGFR 15–29 mL/min/1.73 m^2^ and those with eGFR <15 mL/min/1.73 m^2^ at discharge were lower than that of patients with eGFR ≥60 mL/min/1.73 m^2^ (*p* < .001, *p* < .001 and *p* < .001, respectively, Figure 5).

Multivariate proportional hazard regression analysis identified that age, a preexisting kidney disease, Apache II score, MV, eGFR30–44 mL/min/1.73 m^2^ (HR = 5.46, *p* < .001) and eGFR15–29 mL/min/1.73 m^2^ (HR = 6.76, *p* < .001) and eGFR <15 mL/min/1.73 m^2^ (HR = 23.88, *p* < .001) at discharge were all associated with decreased renal survival (Table 6).

**Table 6.** Multivariate analysis of the variables associated with renal survival (*n* = 403).

Variable	HR (95% CI)	*p*
Age	1.02 (1.00–1.03)	.041*
Gender	0.72 (0.47–1.09)	.115
The cause of AKI		
Sepsis	1.63 (0.87–3.06)	.129
Decreased renal perfusion	1.44 (0.75–2.76)	.275
Surgical	1.40 (0.70–2.78)	.338
Others	–	–
Co-morbidities		
Neoplasm	0.97 (0.56–1.68)	.916
Diabetes	1.56 (0.92–2.64)	.100
Hypertension	0.80 (0.51–1.25)	.326
Pre-CKD	2.07 (1.38–3.11)	<.001*
Apache II score	1.05 (1.02–1.08)	.002*
Saps II score	0.99 (0.98–1.02)	.740
Vasopressors	0.78 (0.51–1.19)	.250
Mechanical ventilation	1.07 (1.03–1.12)	.001*
Renal function at hospital discharge		
eGFR ≥60 mL/min	–	–
eGFR45–59 mL/min	2.64 (0.85–8.15)	.093
eGFR30–44 mL/min	5.46 (2.24–13.29)	<.001*
eGFR15–29 mL/min	6.76 (2.81–16.30)	<.001*
eGFR <15 mL/min	23.88 (10.54–54.11)	<.001*

*Significant at *p* < .05 level.

## Discussion

AKI is the most common complication of critically ill patients and obviously increases rates of in-hospital mortality. This study showed that the in-hospital mortality was 57.2%, coinciding with earlier studies, which reported that an estimated 30–70% of ICU patients suffered from AKI and it resulted in increased in-hospital mortality in critically ill patients requiring dialysis, which accounted for 50–60% [29–31].

Meanwhile, the recent studies indicated that AKI was relevant to long-term outcomes [9–12,32–34]. This study indicated that the 5 year survival rate was 46.3 ± 2.7%, which is similar to the study that the 5 year survival rate was 47% in the followed 226 survivors of RRT in the ICU [35], but is low to the survival rate in the sixth years was 62% in the followed 475 survivors of RRT in the ICU [24].

Recently, some studies have also revealed the relationship between impaired kidney function at discharge and the long-term survival of patients who received RRT in the ICU. In the study conducted by Susanne Stads et al., multivariate analysis demonstrated that an eGFR <30 mL/min/1.73 m^2^ (including patients receiving long-term RRT) at discharge was closely connected with the long-term mortality in 1220 patients [24]. However, in this study eGFR <45 mL/min/1.73 m^2^ was associated with the long-term mortality. The patients with eGFR between 30 and 45 mL/min/1.73 m^2^ also should be paid more attention to. Low eGFR is an important independent risk factor for mortality. This may contribute to the increased inflammation and oxidative stress related to reduced renal function. Besides, kidney dysfunction may be associated with many other physical changes including high levels of homocysteine, hyperuricemia, hypercalcemia and uremia, all of which have detrimental cardiovascular effects [36].

This study also suggested that the long-term renal survival rate was much lower in eGFR <45 mL/min/1.73 m^2^ groups and those groups also had a higher risk of requesting long-term RRT. AKI is now recognized as being in a bidirectional relationship with CKD in hospitalized patients. Preexisting CKD increases the susceptibility for the development of AKI and likewise, exposure to AKI would also accelerate the development of CKD compared with its occurrence on those without AKI [37]. Moreover, AKI in concurrent with CKD leads to ESRD at a higher frequency than AKI alone [38–40]. Bagshaw stated that 10% of critically ill patients with AKI end up with CKD [21]. In another study, the need for RRT increased the likelihood of progression to CKD by 500-fold [22]. Lo et al. [39] researched on 3773 AKI patients (follow-up of 8-years) and found that the proportion of patients with AKI who developed CKD4 and ESRD was 47.9%, which was significantly higher than that in non-AKI group (1.7%). Other studies have also confirmed that AKI is an independent risk factor for the onset of CKD [37–41]. Pre-clinical studies have suggested a multitude of possible mechanisms: acute endothelial injury leading to vascular dropout, nephron loss followed by glomerular hypertrophy or development of fibrosis after sustaining AKI [42].

AKI is not only closely associated with CKD, but also an important cause of CKD, and CKD is also an independent risk factor for AKI patients. Besides, impaired kidney function at discharge is an important risk factor affecting the long-term renal and overall survival of critically ill patients. Therefore, long-term follow-up in the department of nephrology is necessary for patients with an episode of AKI, especially for patients discharged with eGFR <45 mL/min/1.73 m^2^.

This study also indicated that age, causes of AKI (sepsis and decreased renal perfusion), vasopressors were independent risk factors of increased mortality of the objects. This is similar to many studies that have consistently shown that patients who develop ESRD are at higher risk of sepsis, cardiovascular events and surgical operation, and also have a higher mortality risk [43–45]. Moreover, many studies indicated that AKI in association with sepsis was particularly hazardous [46,47]. The mechanism may be that the persistent proinflammatory milieu with an episode of sepsis was amplified by a decline in the ability of the kidneys to clear these toxic molecules. Alternatively, the inflammatory injury to the kidneys may persist and feed a persistent systemic inflammatory phenotype.

This study is a retrospective and observational database study and surely has some limitations. To begin with, objects admitted are restricted to a single center. Furthermore, due to the disadvantage of telephone follow-up, there is no obvious data of renal function. Accepting RRT or kidney transplantation is considered as the only index of renal failure. Some patients who had worse renal outcome without RRT may be missed out during follow-up. Lastly, there were 96 (19.2%) survivors that were out of contact during the follow-up. However, the limitations mentioned are believed to have minimal influence on the results.

## Conclusions

This study showed that impaired kidney function at discharge was shown to be an important risk factor affecting the long-term renal survival rates of critically ill patients with AKI, and a low GFR was an independent risk factor for decreased overall survival.

## Disclosure statement

The authors declare that they have no conflict of interest. This study was approved by the Ethics Committee of The First Affiliated Hospital, Medical College, Zhejiang University.
